# Controllable Synthesis of Zn-Doped α-Fe_2_O_3_ Nanowires for H_2_S Sensing

**DOI:** 10.3390/nano9070994

**Published:** 2019-07-10

**Authors:** Kefeng Wei, Sikai Zhao, Wei Zhang, Xiangxi Zhong, Tingting Li, Baoyu Cui, Shuling Gao, Dezhou Wei, Yanbai Shen

**Affiliations:** 1 Shen Kan Engineering and Technology Corporation, MCC., Shenyang 110169, China; 2 School of Resources and Civil Engineering, Northeastern University, Shenyang 110819, China

**Keywords:** α-Fe_2_O_3_, Zn doping, nanowires, H_2_S, gas sensor

## Abstract

One-dimensional Zn-doped α-Fe_2_O_3_ nanowires have been controllably synthesized by using the pure pyrite as the source of Fe element through a two-step synthesis route, including the preparation of Fe source solution by a leaching process and the thermal conversion of the precursor solution into α-Fe_2_O_3_ nanowires by the hydrothermal and calcination process. The microstructure, morphology, and surface composition of the obtained products were characterized by X-ray diffraction, scanning electron microscopy, transmission electron microscopy, and X-ray photoelectron spectroscopy. It was found that the formation process of α-Fe_2_O_3_ is significantly influenced by the introduction of Zn^2+^. The gas sensing measurements indicated that the sensor based on 1% Zn-doped α-Fe_2_O_3_ nanowires showed excellent H_2_S sensing properties at the optimum operating temperature of 175 °C. Notably, the sensor showed a low H_2_S detection limit of 50 ppb with a sensor response of 1.5. Such high-performance sensing would be ascribed to the one-dimensional structure and high specific surface area of the prepared 1% Zn-doped α-Fe_2_O_3_ nanowires, which can not only provide a large number of surface active sites for the adsorption and reaction of the oxygen and H_2_S molecules, but also facilitate the diffusion of the gas molecules towards the entire sensing materials.

## 1. Introduction

Hydrogen sulfide (H_2_S), as a typical colorless, inflammable, and malodorous gas, is extensively produced in various industrial processes, such as coal mines, water treatment, petroleum refining, and paper industry [1,2]. Specifically, H_2_S is also an extremely toxic gas that poses great threats to human health. For example, it can cause serious damage to human respiratory and nerve systems even at a very low concentration, and the death will occur if H_2_S concentration is higher than 700 ppm [3,4]. According to the criterion set by the American Conference of Government Industrial Hygienists, the threshold H_2_S concentration is only 10 ppm [5]. As well, the Scientific Advisory Board on Toxic Air Pollutants suggests that the acceptable concentration of H_2_S in the atmosphere is 20–100 ppb [6,7]. Therefore, in the perspective of human health protection and environmental monitoring, the selective and reliable H_2_S sensor with the detection limit of ppm and sub-ppm is in urgent demand.

For many years, metal oxide semiconductor (MOS) materials received considerable attention owing to their wide application in the fields of optic [8,9], energy [10,11], catalyst [12,13], and gas sensor [14,15,16]. Specifically in gas sensors, MOS has been considered as the most potential sensing materials in detecting various hazardous gases and has covered most of the monitoring of environmental pollutants [17,18]. With the rapid development of nanoscience and nanotechnology, a large number of nanostructured MOS materials such as ZnO [19], SnO_2_ [20], Fe_2_O_3_ [21], In_2_O_3_ [19], and CuO [22] have been investigated for the application of H_2_S sensing. Among them, Fe_2_O_3_, which is commonly used as the desulfurizer in the purification process of coal gas, is an excellent potential H_2_S sensing material with the outstanding advantages of low cost, good stability, environment friendly, and easy availability [3,23]. 

As we know, the sensing signal of MOS based gas sensors is generated mainly by the adsorption, desorption, and reaction of oxygen and target gases on the surface of the sensing materials [24]. Thus the sensing performance of these sensors is closely related to the microstructure, morphology, crystal size, chemical composition, and synthesis process of the sensing materials [25,26]. On this account, the controllable synthesis of novel types gas sensing materials with different architectures and morphologies have always been the research hotspot in this field. 

Up to now, various α-Fe_2_O_3_ nanostructured materials have been synthesized and investigated for H_2_S sensing. Table 1 gives a comparison between the reported Fe_2_O_3_ based H_2_S sensors. Deng et al. synthesized α-Fe_2_O_3_ nanospheres by a microwave-assisted hydrothermal method. The sensor based on the obtained nanospheres exhibited a peak response of 6 to 10 ppm H_2_S at the operating temperature of 225 °C [27]. Li et al. reported a α-Fe_2_O_3_ nanoparticles based H_2_S sensor. At its optimum operating temperature of 300 °C, the sensor can detect trace H_2_S of 50 ppb with a response of 1.25 while a response of 5.5 to 10 ppm H_2_S is obtained [28]. Li et al. prepared α-Fe_2_O_3_ micro-ellipsoids through a surfactant-free hydrothermal process, and the sensor exhibited a response of 11.7 to 100 ppm H_2_S at 350 °C [29]. Furthermore, the effect noble metals, which are effective catalysts to improve the gas sensing properties of semiconductor gas sensing materials, on the H_2_S sensing performance of α-Fe_2_O_3_ based sensors were also extensively investigated. Wang et al., prepared Pt, Pd, and Ag doped α-Fe_2_O_3_ nanoparticles and systematically compared their H_2_S sensing characteristics with pure α-Fe_2_O_3_ nanoparticles. It had been found that the introduction of Pt, Ag, and Pd not only can increase the H_2_S response, but also can decrease the optimum operating temperature of the sensors [30,31,32]. Balourian et al. studied the H_2_S sensing properties of Au functionalized α-Fe_2_O_3_ thin films. The results demonstrated that the sensor based on 2.33% Au modified α-Fe_2_O_3_ thin films exhibited the highest H_2_S response. The optimum operating temperature of which was 250 °C with a response of 6.4 to 10 ppm H_2_S. It also found that the response time of this sensor was very long (27 min) [33]. Despite these progress have been made, as can be seen in Table 1, it can be found that there are still some limitations to meet the requirements of practical application, including relatively low sensitivity, high detection limit, and high operating temperature. In addition, the long recovery time is also a critical problem for H_2_S detection.

Owing to their high surface to volume ratio, good stability, and fast mass transport, one-dimensional nanostructures (e.g., nanorod, nanowire, nanofiber, and nanotube) are found to be an efficient architecture for the application of high-performance gas sensing [34,35]. Therefore, in this paper, Zn-doped α-Fe_2_O_3_ nanowires were controllably synthesized by using the pure pyrite as the source of Fe element, and it was found that Zn^2+^ took a vital part in the formation process of α-Fe_2_O_3_ one-dimensional structure. Furthermore, the microstructure, morphology, composition, and H_2_S sensing performance of the synthesized products were systematically studied.

## 2. Materials and Methods

### 2.1. Materials

The pure pyrite powders with a high grade of approximately 99% and the particle size of smaller than 74 μm were obtained from Gongchangling mineral company, Anshan, China. Zinc chloride (ZnCl_2_) and sodium hydroxide (NaOH) were analytical grade and purchased from Sinopharm Chemical Reagent Co., Ltd., Shenyang, China. Hydrochloric acid (HCl) was purchased from Kemiou Reagent Co., Ltd., Tianjin, China. All the reagents were directly used as received without further purification.

### 2.2. Preparation of Fe Source Solution

**Figure 1:**
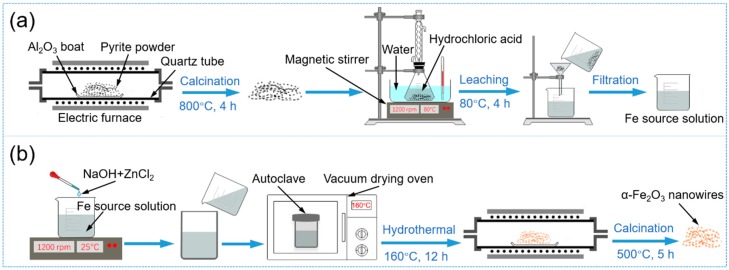
Schematic diagram of (**a**) the preparation process of Fe source solution and (**b**) preparation process of Zn-doped α-Fe_2_O_3_ nanowires.

The Fe source solution that used to synthesis α-Fe_2_O_3_ nanowires was obtained from pure pyrite by a leaching process. In a typical procedure as shown in Figure 1a, 0.3 g pure pyrite powders were placed in an Al_2_O_3_ boat and then calcined in air at 800 °C for 4 h in a tubular furnace with the heating rate of 10 °C/min. The obtained samples were dissolved in 60 mL HCl (1.4 M) while being stirred at 80 °C for 4 h. Then, the insoluble impurities were removed by filtration, and the Fe source solution with the Fe concentration of 19.9 g/L was obtained.

### 2.3. Synthesis of Zn-Doped α-Fe_2_O_3_ Nanowires

A typical procedure for synthesizing Zn-doped α-Fe_2_O_3_ nanowires was schematically illustrated in Figure 1b and carried out as follows. A pre-defined amount of ZnCl_2_ solution (0.035 M) was added in 20 mL the as-prepared Fe source solution. The pH was adjusted to 13 by the dropwise addition of NaOH solution (3 M) under constant magnetic stirring for 30 min at room temperature. The solution was then transferred into a 200 mL Teflon-lined autoclave, hydrothermal reacted at 160 °C for 12 h, and cooled down to room temperature naturally. The resulted precipitates were washed by centrifuging-washing cycles with distilled water and ethanol followed by drying at 60 °C for 4 h. Finally, the dried samples were calcined at 500 °C in air for 5 h. The 1% and 3% Zn-doped α-Fe_2_O_3_ were estimated by calculating the molar ratio of Zn/Fe in the precursor solution.

### 2.4. Characterizations

The crystalline phase of the prepared products was analyzed by X-ray diffractometer (XRD, PANalytical X’Pert Pro, Cu K_α_ radiation, λ = 1.5406 Å, PANalytical B.V., Almelo, Overijssel, Netherlands). Data was collected in the 2θ range of 20–80°. Their morphology and microstructure were characterized by a S-4800 scanning electron microscope (SEM, HITACHI, Tokyo, Japan), which was operated at 2 kV. The transmission electron microscopy (TEM), high-resolution TEM transmission electron microscopy (HRTEM), and selective area electron diffraction (SEAD) images were conducted on JEM-2100F microscope (JEOL, Tokyo, Japan) with the operating voltage of 200 kV. The surface component and bonding state analysis were performed on an X-ray photoelectron spectroscope (XPS, Thermo Scientific Escalab 250Xi, Thermo Fisher Scientific, Waltham, MA, USA) with Al K_α_ radiation (1486.6 eV).

### 2.5. Fabrication and Measurement of Gas Sensor

**Figure 2:**
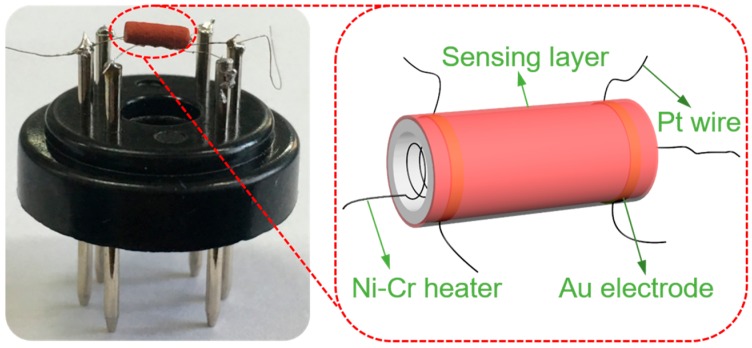
Structure schematic of a typical gas sensor.

The photo and schematic in Figure 2 illustrated the gas sensor structure. For the fabrication of the sensor, a proper amount of the as-prepared Zn-doped α-Fe_2_O_3_ nanowires were dispersed in ethanol with the assistance of ultrasonication to form the sensing paste. Then, the sensing paste was coated onto the outer surface of an Al_2_O_3_ tube (1.2 mm in external diameter, 0.8 mm in internal diameter, 4 mm in length) by using a brush. Electric contacts were achieved by a pair of gold electrodes, on each of which a pair of platinum wires were connected. The operating temperature of the sensor was controlled by a Ni-Cr coil that inserted through the coated alumina tube. In order to improve the thermal stability, the sensor was aged at 300 °C in air for two days before testing. The gas sensing performance of the sensor was measured on WS-30A commercial static test system (Winsen Electronics Science and Technology Co., Ltd., Henan, China). The sensor response (*S*) is defined here as the ratio of the sensor resistance in fresh air (*R*_a_) to that in target gas (*R*_g_) under static conditions; the values do not represent steady-state dynamical equilibrium conditions, as would be the case in a flow-through measuring setup. As well, the response and recovery times are defined as the time for the sensor resistance to achieve 90% variation after H_2_S is injected and removed, respectively.

## 3. Results and Discussion

### 3.1. Structural and Morphology Characterization

**Figure 3:**
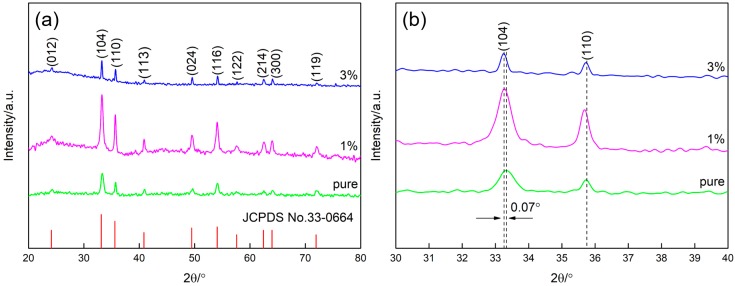
XRD patterns of pure α-Fe_2_O_3_, 1% Zn-doped α-Fe_2_O_3_, and 3% Zn-doped α-Fe_2_O_3_ in the 2θ range of (**a**) 20–80° and (**b**) 30–40°.

Figure 3 displays the typical XRD patterns of the synthesized samples with different molar ratios of Zn/Fe. As can be seen in Figure 3a, the diffraction peaks of all the samples can be well indexed to the hexagonal α-Fe_2_O_3_ phase with unit cell parameters of a = b = 5.0356 Å and c = 13.7489 Å (JCPDS card No. 33-0664). The absence of the diffraction peaks corresponding to Zn in the patterns may be ascribed to the low content of Zn. Additionally, no characteristic peaks from other impurities are detected, indicating that all the products are of high phase purity. Furthermore, it can be observed in Figure 3b that the diffraction peaks of 1% and 3% Zn-doped α-Fe_2_O_3_ have a slight shift to lower value compared with pure α-Fe_2_O_3_. These shifts might be the result that Zn^2+^ is incorporated into the crystal lattice of α-Fe_2_O_3_ leading to the slight change of the crystal structure.

**Figure 4:**
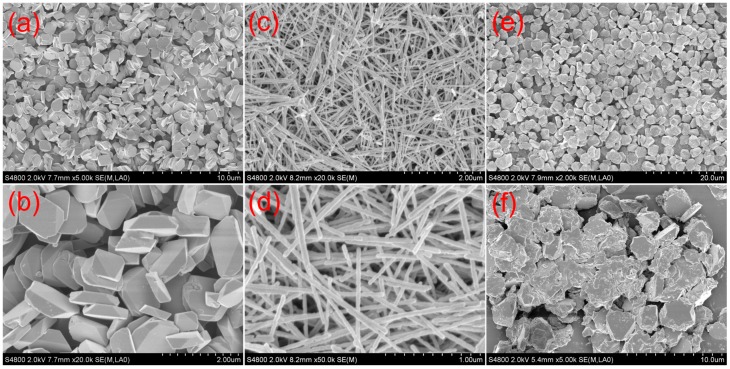
SEM images of α-Fe_2_O_3_ prepared with different Zn/Fe molar ratios. (**a**,**b**) are pure α-Fe_2_O_3_. (**c**,**d**) are 1% Zn-doped α-Fe_2_O_3_. (**e**,**f**) are 3% Zn-doped α-Fe_2_O_3_.

The SEM images of the obtained α-Fe_2_O_3_ with different Zn/Fe molar ratios is presented in Figure 4. It can be clearly observed that Zn^2+^ plays a vital role in controlling the microstructure of α-Fe_2_O_3_. As can be seen in Figure 4a, α-Fe_2_O_3_ that prepared without Zn is plate-like with a smooth surface, and its thickness is nearly 300 nm. For 1% Zn-doped α-Fe_2_O_3_ sample, highly dispersive nanowires with the diameter of ~50 nm and the length of 2–3 μm are obtained, indicating that Zn^2+^ can significantly promote α-Fe_2_O_3_ to form one-dimensional structure. In addition, it can be found that 1% Zn-doped α-Fe_2_O_3_ nanowires exhibit a coarser surface than pure α-Fe_2_O_3_ and some pores on their surface can be clearly observed. However, the structure of α-Fe_2_O_3_ becomes plate-like again as the doping percentage of Zn increases to 3%. Moreover, the surface of these plates is coarser while the dispersity is apparently deteriorated compared with pure α-Fe_2_O_3_. In summary, the formation process and the morphology of α-Fe_2_O_3_ are closely related to the molar ratio of Zn/Fe in the precursor solution, and the homogeneous α-Fe_2_O_3_ nanowires can be controllably synthesized by introducing a proper amount of Zn^2+^. It is worthy to mention that the effect of some other inorganic salt ions such as In^3+^, Ni^2+^, Cu^2+^, and Mg^2+^ on the morphology of the final α-Fe_2_O_3_ have also been investigated, and it is interestingly found that α-Fe_2_O_3_ nanowires can only be obtained in the presence of Zn^2+^.

**Figure 5:**
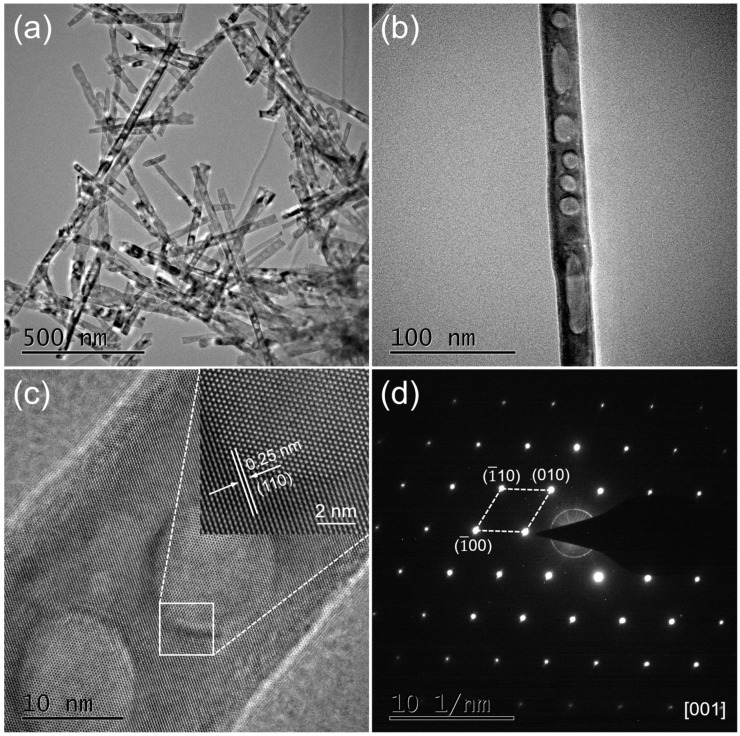
(**a**) High-magnification and (**b**) low-magnification TEM images of 1% Zn-doped α-Fe_2_O_3_ nanowires. (**c**) HRTEM image of 1% Zn-doped α-Fe_2_O_3_ nanowires. (**d**) SAED pattern of 1% Zn-doped α-Fe_2_O_3_ nanowires.

TEM measurements were used for further characterizations of the prepared 1% Zn-doped α-Fe_2_O_3_ nanowires. As shown in Figure 5a, the obtained products are wire-like with a diameter of about 50 nm, which is associated with the results of SEM characterization. Also, the irregular pores as observed in SEM measurement can be apparently found in Figure 5b. However, it can be assumed from Figure 5c that the pores seemly only exist on the surface of the α-Fe_2_O_3_ nanowires with a certain depth, and they don’t penetrate through the whole nanowires. The measured lattice spacing between two adjacent fringes is 0.25 nm, which corresponds to the (110) plane of hexagonal structured α-Fe_2_O_3_. The corresponding SAED pattern in Figure 5d, which is indexed to [001] zone-axis, reveals that as-prepared 1% Zn-doped α-Fe_2_O_3_ nanowires are of single crystal.

**Figure 6:**
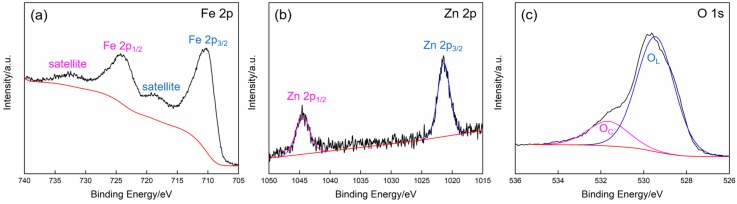
XPS spectra of 1% Zn-doped α-Fe_2_O_3_ nanowires. (**a**) Fe 2p spectrum. (**b**) Zn 2p spectrum. (**c**) O 1s spectrum.

For further illustration of the surface composition and chemical states of 1% Zn-doped α-Fe_2_O_3_ nanowires, the XPS spectra were studied. Figure 6a shows the narrow scan spectrum of Fe 2p. The two dominant peaks located at 710.3 and 724.6 eV are indexed to Fe 2p_3/2_ and Fe 2p_1/2_ peaks of α-Fe_2_O_3_, respectively. In addition, on the respective higher binding energy sides of Fe 2p_3/2_ and Fe 2p_1/2_ peaks at about 8 eV (718.2 and 732.8 eV), the broad satellite peaks can be clearly observed, which further confirms that Fe species in the as-prepared samples exist in the form of α-Fe_2_O_3_ phase [35,36]. The high-resolution spectrum of Zn 2p is displayed in Figure 6b, in which the two distinct peaks at 1021.4 and 1044.5 eV with the binding energy separation value of 23.1 eV are in good accordance with Zn 2p_3/2_ and Zn 2p_1/2_, respectively, demonstrating the normal chemical state of Zn^2+^ in the obtained products [37,38]. Furthermore, from the report of the XPS analysis results, it can be found that the molar ratio of Zn/Fe is 10%, which is much higher than that in the precursor solution, indicating that the doped Zn element is mainly distributed in the surface region of the finally prepared Zn-doped α-Fe_2_O_3_. The high-resolution spectrum of O 1s core-level with an obvious shoulder in Figure 6c is resolved to two Gaussian function peaks with binding energies centered at 529.5 and 531.8 eV, corresponding to the lattice oxygen species (O_L_) and the chemisorbed oxygen species (O_C_), respectively [39,40].

### 3.2. Growth Mechanism

**Figure 7:**
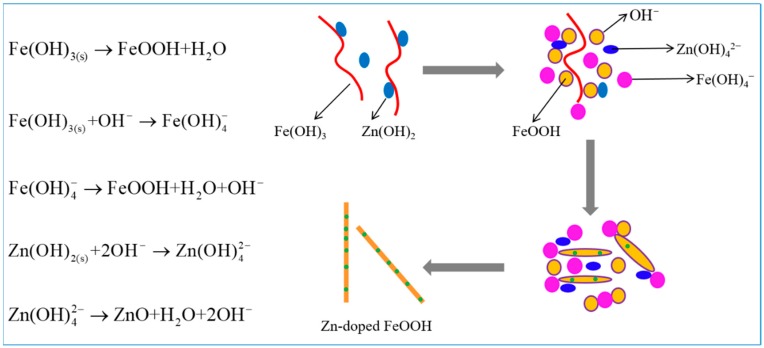
Schematic diagram of the formation process of Zn-doped α-Fe_2_O_3_ nanowires.

There are two steps for the growth of the Zn-doped α-Fe_2_O_3_ nanowires including the nucleation and their anisotropic growth (Figure 7). Figures S1 and S2 give the calculated distribution diagram of iron ion and zinc ion solutions as a function of pH, respectively. As can be seen in these figures, the Fe^3+^ will react with OH^−^ prior to Zn^2+^ to form the flocculent precipitation of amorphous Fe(OH)_3_ when increasing the pH of the precursor solution by dropwise addition of NaOH solution. During this process, a part of Zn^2+^ is co-precipitated with Fe^3+^ and incorporated in amorphous Fe(OH)_3_, while the others is precipitated from the precursor solution in the form of Zn(OH)_2_ with the further increase of pH. For the hydrothermal process, because of the high temperature, pressure, and alkaline condition, the colloidal Fe(OH)_3_ is dehydronated and dissolved gradually followed by a nucleation and crystallization process to form α-(Fe, Zn)OOH. As reported previously [41], the existence of Zn^2+^ can improve the stability of the Fe(OH)_3_, thus the dehydration process can be carried out steadily without too fast partial reaction. On the other hand, it can be concluded that a proper amount of Zn^2+^ can effectively induce the formation of one-dimensional structured α-FeOOH. This phenomenon is similar to the observation of Stjepko Krehula and needs further investigation [42]. Finally, the Zn-doped α-Fe_2_O_3_ nanowires can be obtained by the calcination treatment.

### 3.3. Gas Sensing Properties

**Figure 8:**
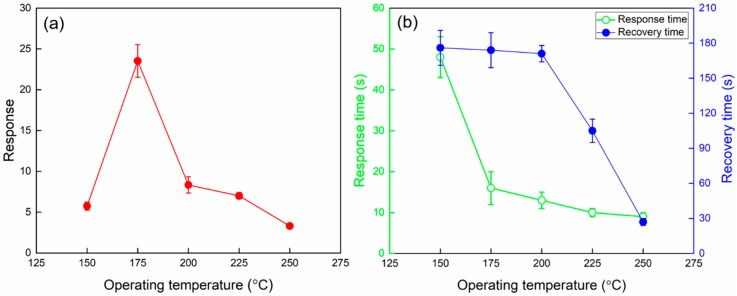
(**a**) Response and (**b**) response and recovery times of 1% Zn-doped α-Fe_2_O_3_ nanowires upon exposure to 5 ppm H_2_S at different operating temperatures.

Considering that the operating temperature of the MOS based gas sensors has a significant influence on their gas sensing performance. The response and response/recovery times of the sensor based on 1% Zn-doped α-Fe_2_O_3_ nanowires towards 5 ppm H_2_S were firstly examined at different operating temperatures. As can be seen in Figure 8a, the sensor response is significantly improved while the operating temperature increases from 150 to 175 °C. The maximum sensor response of 23.5 is obtained at the operating temperature of 175 °C. And the apparent downward trend can be observed with further increasing the operating temperature. At low operating temperatures, there is no sufficient active energy for H_2_S molecules to react with the oxygen species that chemisorbed on the surface of 1% Zn-doped α-Fe_2_O_3_ nanowires, resulting in low response. While the sensor response tends to decrease at the temperature higher than the optimum is mainly caused by the increased desorption rate of H_2_S and oxygen molecules [43]. As presented in Figure 8b, both the response and recovery times are reduced dramatically with the increase of the operating temperature, which is mainly caused by the faster adsorption and desorption kinetics of the gases at high operating temperature. At the optimum operating temperature of 175 °C, the fast response time of 16 s and recovery time of 174 s for 1% Zn-doped α-Fe_2_O_3_ nanowires to 5 ppm H_2_S are obtained.

**Figure 9:**
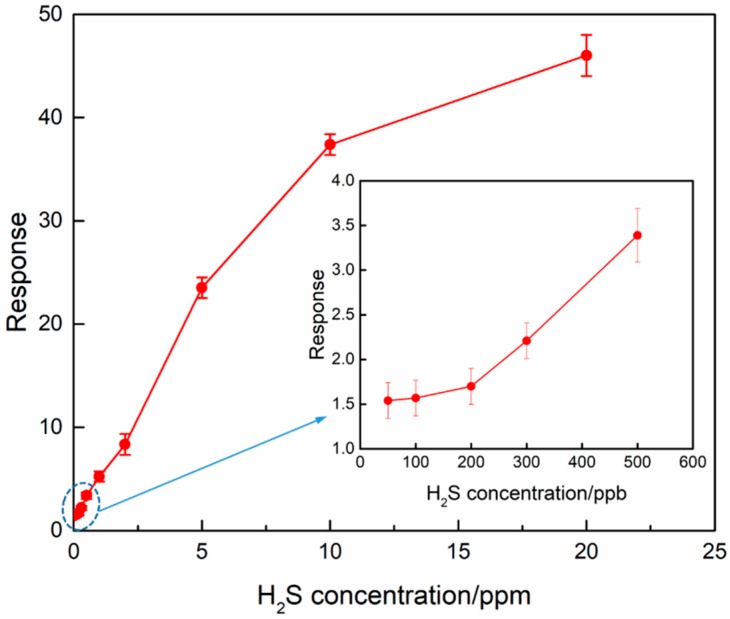
Responses of 1% Zn-doped α-Fe_2_O_3_ nanowires upon exposure to various H_2_S concentrations at the operating temperature of 175 °C.

Figure 9 displays the response of the sensor based on 1% Zn-doped α-Fe_2_O_3_ nanowires as a function of H_2_S concentration at 175 °C. It is very clear that the sensor response shows an obvious H_2_S concentration-dependent feature and increases monotonically with the increase of H_2_S concentration in the range of 50 ppb–10 ppm, and then changes slightly as further increasing H_2_S concentration. Such phenomena can be explained as follows. At relatively low H_2_S concentrations, the surface reaction is enhanced with the increase of H_2_S concentration, leading to a remarkable and linear increase of the sensor response. However, for MOS gas sensing materials, the number of the surface active site of the sensing materials is almost constant at a certain operating temperature. Therefore, the sensor response would gradually become saturated with the further increase of H_2_S concentration for the reason that there are no more active sites available for the adsorption and reaction of H_2_S molecules [44,45]. Specifically, it is worth mentioning that the sensor still exhibits a notable response of 1.5 at a relatively low H_2_S concentration of 50 ppb, indicating a promising application potential in the monitoring of trace amount of H_2_S.

**Figure 10:**
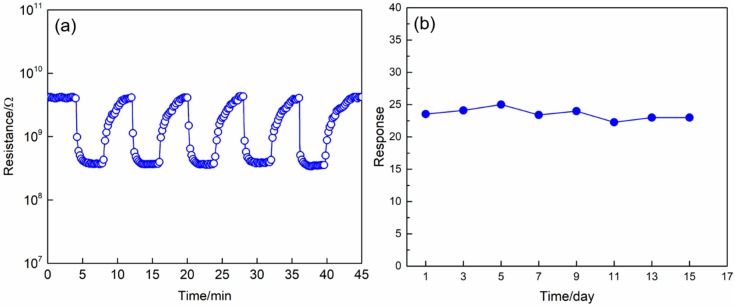
(**a**) Dynamic response and recovery curves of 1% Zn-doped α-Fe_2_O_3_ nanowires upon exposure to 5 ppm H_2_S for five cycles at the operating temperature of 175 °C. (**b**) Long-term stability of the sensor based on 1% Zn-doped α-Fe_2_O_3_ nanowires upon exposure to 5 ppm H_2_S at the operating temperature of 175 °C.

The reproducibility and stability are of great importance parameters to evaluate the performance of gas sensors. Figure 10a presents the dynamic response characteristics of 1% Zn-doped α-Fe_2_O_3_ nanowires upon exposure to 5 ppm H_2_S for five cycles at 175 °C. Apparently, the resistance of the sensor decreases abruptly upon the injection of H_2_S, following which it increases rapidly and recovers to its initial value once H_2_S is removed. This sensing behavior reveals the n-type semiconductor conduction characteristic of the prepared 1% Zn-doped α-Fe_2_O_3_ nanowires. Furthermore, the dynamic response and recovery processes are almost the same without a clear change upon five successive sensing measurement cycles to the same H_2_S concentration of 5 ppm, demonstrating the excellent reversibility and reproducibility of the 1% Zn-doped α-Fe_2_O_3_ nanowires based sensor when alternately exposed to air and H_2_S. Figure 10b gives the long-time stability of the sensor at the optimal operating temperature of 175 °C, as can be seen in this figure, the sensor response to 5 ppm H_2_S only showed a small fluctuation in 15 days, which indicates the excellent stability of the sensor.

**Figure 11:**
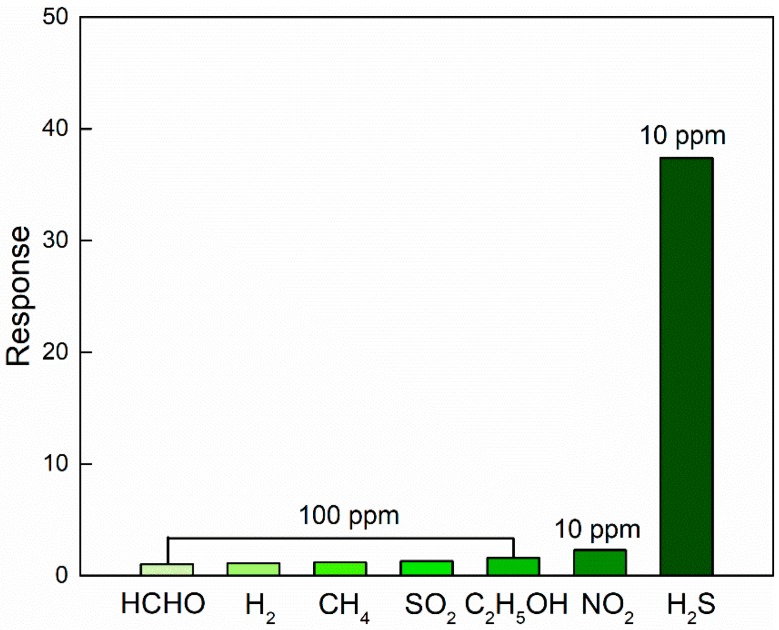
Responses of 1% Zn-doped α-Fe_2_O_3_ nanowires upon exposure to different gases at the operating temperature of 175 °C.

In order to recognize the specific target gas in a multicomponent gas environment, an excellent selectivity is required for high-performance gas sensors. The response of the sensor upon exposure to seven typical gases at 175 °C were examined to determine the sensor selectivity, including formaldehyde (HCHO), hydrogen (H_2_), methane (CH_4_), sulfide dioxide (SO_2_), ethanol (C_2_H_5_OH), nitrogen dioxide (NO_2_) and hydrogen sulfide (H_2_S), and the results are presented in Figure 11. In detail, the sensor responses are 1.0, 1.1, 1.2, 1.3, 1.6, 2.3, and 37.4 to 100 ppm HCHO, 100 ppm H_2_, 100 ppm CH_4_, 100 ppm SO_2_, 100 ppm C_2_H_5_OH, 10 ppm NO_2_, and 10 ppm H_2_S, respectively. Such observations reveal the excellent H_2_S selectivity of the present sensor over other gases at 175 °C.

As for MOS based gas sensors, it is well known that the detection of target gas depends on the conductivity changes of the sensing materials that mainly caused by the adsorption and desorption of oxygen species on their surface [46,47]. Therefore, the sensing mechanism of α-Fe_2_O_3_ nanowires can be explained by the reaction between chemisorbed oxygen species and H_2_S molecules. When the sensor based on 1% Zn-doped α-Fe_2_O_3_ nanowires is in ambient air, the oxygen molecules are diffused and adsorbed on its surface, followed by transferring into O^−^, O^2−^, or O_2_^−^ by capturing electrons from the conduction band, which results in the decrease of the electron concentration and the formation of the depletion layer in the surface region of α-Fe_2_O_3_ nanowires. As a consequence, the sensor is in high-resistance state. When the sensor is exposed to H_2_S, H_2_S molecules react with the chemisorbed oxygen species on the surface of α-Fe_2_O_3_ nanowires and as a result the captured electrons are released back to the conduction band, which narrows the depletion layer and eventually results in a remarkable decrease of the sensor resistance. It can be representatively expressed by:

H_2_S + 3O^2−^ → H_2_O + SO_2_ + 6e^−^ (1)

In addition, the H_2_S gas molecules can also react with α-Fe_2_O_3_ as follows:

3H_2_S + Fe_2_O_3_ → Fe_2_S_3_ + 3H_2_O (2)

Fe_2_S_3_ → FeS + FeS_2_ (3)

Thus, the iron sulphides will be formed on the surface of the α-Fe_2_O_3_ nanowires, which will also increase the conductivity of the sensor because of the low band gap intrinsic characteristic of such iron sulphides. However, as reported by Singh [33], the reaction (1) plays a more predominant role in the sensing process.

Once the sensor breaks away from the H_2_S atmosphere, α-Fe_2_O_3_ nanowires would be covered by oxygen species again, and as a result the sensor recovers to its initial state. The excellent sensing properties of α-Fe_2_O_3_ nanowires could be mainly ascribed to the unique one-dimensional structure. On the one hand, it can provide a large accessible surface area and thus a large number of available surface active sites for the sensing reaction between H_2_S molecules and chemisorbed oxygen species. On the other hand, it can facilitate the diffusion of the gas molecules towards the entire sensing materials.

## 4. Conclusions

An efficient route was developed for the preparation of Zn-doped α-Fe_2_O_3_ nanowires with a large specific surface area. The pure pyrite was employed as the source of Fe, and Zn^2+^ was introduced to induce the formation of one-dimensional structure of α-Fe_2_O_3_. Interestingly, α-Fe_2_O_3_ nanowires can only be obtained when the molar ratio of Zn/Fe is 1% in the precursor solution. The synthesized Zn-doped α-Fe_2_O_3_ nanowires are single crystal hexagonal structure with the diameter and length of ~50 nm and 2–3 μm, respectively. The H_2_S sensor was fabricated by using 1% Zn-doped α-Fe_2_O_3_ nanowires as the sensing material. At the optimum operating temperature of 175 °C, the sensor exhibited a high response of 23.5 to 5 ppm H_2_S with fast response time of 16 s and recovery time of 171 s. Dramatically, the detection limit of 1% Zn-doped α-Fe_2_O_3_ nanowires based sensor was found to be as low as 50 ppb with a remarkable response of 1.5. Furthermore, the sensor also showed excellent reversibility, reproducibility, selectivity, and stability, indicating that the as-prepared Zn-doped α-Fe_2_O_3_ nanowires can be a promising H_2_S sensing material.

## Figures and Tables

**Table 1:** Comparison between different α-Fe_2_O_3_ based H_2_S sensors. (C(H_2_S): H_2_S concentration; T_op_: operating temperature of the sensor; T_res_/T_rec_: response time/recovery time; DL: detection limit; Ref: references).

Sensing Materials	C(H_2_S)	T_op_	R	T_res_/T_rec_	DL (ppm)	Ref
α-Fe_2_O_3_ nanospheres	10	228	6	52 s/-	1 ppm	[27]
α-Fe_2_O_3_ nanoparticles	10	300	5.5	30/5 s	0.05	[28]
α-Fe_2_O_3_ micro-ellipsoids	10	350	3	80/7 s (0.5 ppm)	0.5	[29]
Pt:α-Fe_2_O_3_	10	160	147.5	-/-	-/-	[30]
Pd:α-Fe_2_O_3_	10	160	46.6	-/-	10	[31]
Ag:α-Fe_2_O_3_	100	160	220	42/26 s	60	[32]
Au:α-Fe_2_O_3_	10	250	6.4	1.65/27 min	1	[33]
α-Fe_2_O_3_ nanowires	5	175	23.5	16/174 s	0.05	This work

## Supplementary Materials

The following are available online at https://www.mdpi.com/2079-4991/9/7/994/s1, Figure S1: Concentration logarithmic diagram of Fe^3+^ hydrolysis components (Fe^3+^: 0.14 mol/L), Figure S2: Concentration logarithmic diagram of Zn^2+^ hydrolysis components (Zn^2+^: 1.4 × 10^−3^ mol/L).
