# Supplementary material for: Controllable Synthesis of Zn-Doped α-Fe2O3 Nanowires for H2S Sensing
Source: Nanomaterials (Basel). 2019 Jul 10;9(7):994. doi: 10.3390/nano9070994 (PMC6669516; doi:10.3390/nano9070994)
Supplement: Supplementary file 1 [file nanomaterials-09-00994-s001.pdf]

Supplementary Information

Controllable Synthesis of Zn-Doped α-Fe_2O_3 Nanowires for H_2S Sensing

Kefeng Wei [1], Sikai Zhao [2,*], Wei Zhang [2], Xiangxi Zhong [2] and Tingting Li [2], Baoyu Cui [2], Shuling Gao [2], Dezhou Wei [2] and Yanbai Shen [2,*]

[1] Shen Kan Engineering and Technology Corporation, MCC., Shenyang, 110169, China

[2] School of Resources and Civil Engineering, Northeastern University, Shenyang, 110819, China

* Correspondence: zhaosikai@stumail.neu.edu.cn (S.Z.); shenyanbai@mail.neu.edu.cn (Y.S.); Tel.: +86-24-8368-7381 (Y.S.)

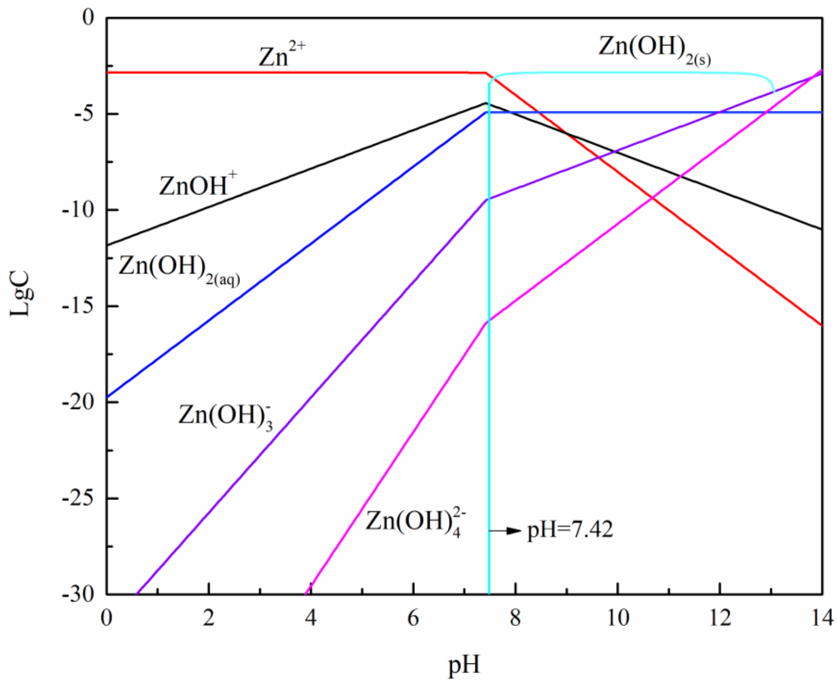

Figure S1. Concentration logarithmic diagram of Fe^{3+} hydrolysis components (Fe^{3+}: 0.14mol/L).

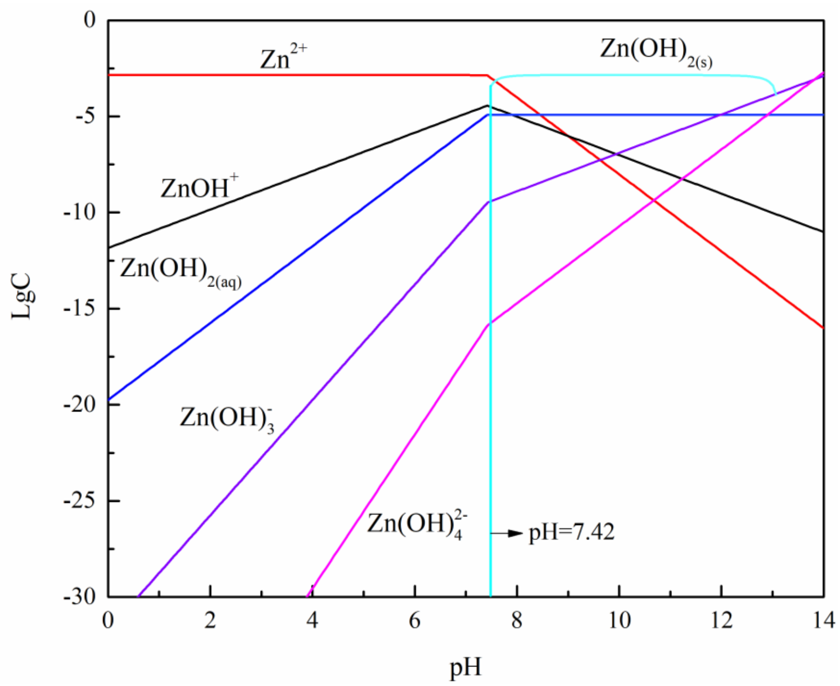

Figure S2. Concentration logarithmic diagram of Zn^{2+} hydrolysis components (Zn^{2+}: 1.4×10^{-3} mol/L).
